# Substituting Chromium in Iron-Based Catalysts for the High-Temperature Water–Gas Shift Reaction

**DOI:** 10.1021/acscatal.2c03871

**Published:** 2022-10-27

**Authors:** M. I. Ariëns, L.G.A. van de Water, A. I. Dugulan, E. Brück, E.J.M. Hensen

**Affiliations:** † Fundamental Aspects of Materials and Energy, Delft University of Technology, Mekelweg 15, 2629 JB Delft, The Netherlands; ‡ Laboratory of Inorganic Materials and Catalysis, Department of Chemical Engineering and Chemistry, Eindhoven University of Technology, P.O. Box 513, 5600 MB Eindhoven, The Netherlands; § Johnson Matthey, P.O. Box 1, Belasis Avenue, Billingham, Cleveland TS23 1LB, United Kingdom

**Keywords:** water−gas shift reaction, iron oxide, chromium replacement, Mössbauer spectroscopy, industrially relevant conditions

## Abstract

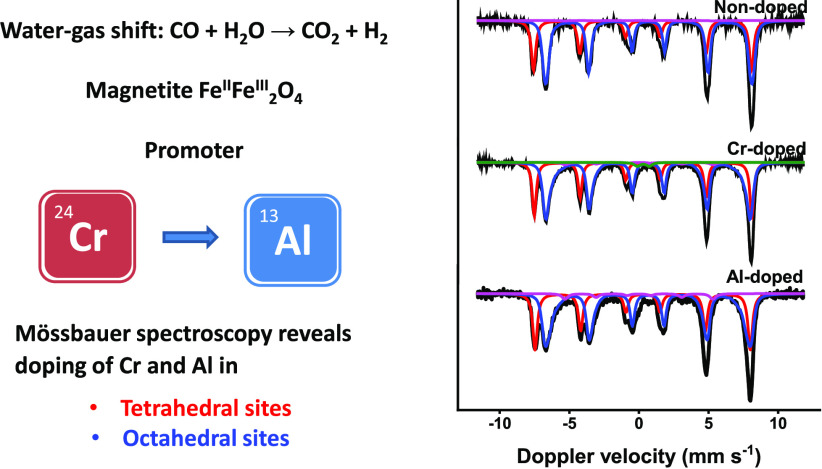

A set of doped iron oxides (chromium, aluminum, gallium, indium, manganese, zinc, niobium) were prepared by a one-step coprecipitation/calcination approach evaluated for their WGS activity under industrially relevant conditions and characterized in detail. The WGS activity after ageing the doped catalyst for 4 days at 25 bar follows the order chromium ≈ aluminum > gallium > indium > manganese > zinc > niobium for copper-codoped catalysts. The activated catalysts predominantly consist of magnetite, irrespective of the dopant. Mössbauer spectra of aged catalysts showed that aluminum and zinc occupy both tetrahedral and octahedral sites of magnetite, while chromium, gallium, indium, manganese, and niobium preferentially substitute octahedral iron. The incorporation of trivalent metal ions of similar size to octahedral Fe^3+^ (i.e., chromium, aluminum, gallium) results in moderate to high CO conversion, irrespective of incorporation in tetrahedral or octahedral sites. The substitution of Fe^2+^ with Mn^2+^ results in an increased Fe^3+^/Fe^2+^ ratio. Incorporation of Zn^2+^ in tetrahedral sites (replacing Fe^3+^ ions) leads to a complex structure where the charge balance is compensated from the octahedral sites. Separate dopant metal oxide phases were observed in indium- and niobium-doped catalysts. XPS shows that copper is present as a separate phase in activated copper-codoped catalysts. Aluminum is identified as the most promising promoter for substituting chromium in commercial high-temperature WGS catalysts on the basis of their similar high CO conversion although incorporation of these dopants into the magnetite structure differed substantially.

## Introduction

Approximately 80% of industrial hydrogen gas is produced from natural gas by steam reforming, followed by the water–gas shift (WGS) reaction 1.^1^ The industrial WGS process involves two temperature stages to optimize CO conversion. High-temperature (water–gas) shift (HTS) removes the bulk of CO from the gas stream at temperatures between 350 and 450 °C, while low-temperature (water–gas) shift (LTS) removes most of the remaining CO at 190–250 °C.^1−4^
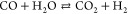
1The active HTS catalyst, the mixed-valence iron oxide compound magnetite (Fe_3_O_4_), is obtained upon partial reduction of the Fe^3+^-oxide/oxyhydroxide precursor species under WGS conditions.^5,6^ Chromium and copper are used as promoters in commercial HTS catalysts to enhance the stability and activity. Chromium exists as Cr^6+^ and Cr^3+^ in the calcined catalyst precursor and ends up as Cr^3+^ in the active magnetite phase.^7^ Chromium doping limits Fe^2+^ formation during activation, resulting in a partially oxidized magnetite structure,^5,8^ thereby improving the thermal stability and preventing overreduction of the active phase. Copper is known to facilitate the partial reduction of Fe^3+^-oxide/oxyhydroxide during magnetite formation, and it has also been shown that the presence of Cu^0^ nanoparticles on the surface of the active magnetite catalyst, partially covered by an iron oxide layer, results in additional active sites.^9,10^

Tightening regulations on hazardous chemicals call for replacement of chromium by an effective alternative with reduced environmental impact.^11^ This has motivated researchers to identify dopants that can replace chromium in HTS catalysts.^12,13^ Comprehensive reviews on alternatives for chromium can be found in the literature.^2,14^ A direct comparison of the potential of these alternative dopants is difficult because the catalyst preparation methods and specific preparation details, such as the calcination temperature, doping levels, as well as test conditions (temperature, pressure, feed gas composition, and test duration), vary among such investigations. The replacement of chromium by aluminum has received most attention.^14^ Zhu et al. showed that aluminum- and aluminum–copper-doped catalysts show comparable thermal stability as chromium-doped catalysts during the WGS reaction at different temperatures at 1 bar for 90 min.^12^ Meshkani and Rezaei^15^ observed that an aluminum-doped HTS catalyst with an aluminum/iron ratio of 1:10 at/at and an atomic copper/iron ratio of 1:10 exhibited a higher conversion than a commercial catalyst after 2 h in a WGS test at 1 bar. It should be mentioned that practical catalysts have typical lifetimes of several years in commercial operation.^4^ Natesakhawat et al.^16^ showed that aluminum doping prevents sintering of magnetite during the WGS reaction. Moreover, they found that the effect of copper promotion depended on the preparation method, as reported before for chromium-doped catalysts.^17^ Cerium-doped iron oxide catalysts have also been thoroughly investigated in recent years.^13,15,18,19^ Smirniotis’ group^20^ showed that chromium- and cerium-codoped catalysts were the most active in the WGS reaction at atmospheric pressure among a series of chromium-, cerium-, manganese-, cobalt-, nickel-, copper-, and zinc-doped catalysts. A sample doped with cerium and chromium in an equimolar ratio provided the highest WGS activity.^21^ However, codoping of the cerium-doped catalyst with copper led to faster deactivation due to overreduction of the active phase to FeO.^19^ Meshkani and Rezaei, however, found that a cerium-doped catalyst had the lowest CO conversion among a series of cerium-, manganese-, aluminum-, and chromium-doped catalysts.^15^ It is worthwhile to mention that also catalysts codoped with nickel have been explored, although the presence of other promoters like sodium^22^ or niobium^23^ is required to suppress methanation.

The local structure of iron oxide HTS catalysts modified with alternative dopants has not been investigated systematically.^12,15^ Another limitation of earlier studies is that most of the activity tests were carried out at atmospheric pressure for a relatively short period under conditions different from those used in commercial HTS configurations.^4^ Our previous study^5^ showed that Cr^3+^ is incorporated in the octahedral sites of magnetite in the activated catalyst and that its presence in the fresh catalyst limits the formation of Fe^2+^ during the activation procedure. The incorporation of copper in the magnetite structure was found to be unlikely,^6^ which is confirmed by the presence of a separate Cu^0^ phase in the form of nanoparticles.^9,10^ In this study, we investigate the potential of alternative dopants to replace chromium (aluminum, gallium, indium, zinc, manganese, and niobium). All catalysts were prepared with and without codoping with copper. In addition to screening these novel catalysts for their WGS activity under industrially relevant conditions, a thorough investigation of the catalyst structure was made after ageing for 4 days at 25 bar. The group 13 elements aluminum, gallium, and indium were chosen to evaluate the effect of the octahedral ionic radius of trivalent dopants on the local structure. Zinc, manganese (2+), and niobium were selected to investigate the effect of elements with different oxidation states. In addition to routine characterization techniques such as XRD and XPS, Mössbauer spectroscopy was employed to investigate the local structure of the activated promoted magnetite catalysts. Mössbauer spectroscopy is a highly sensitive technique for bulk-iron species, capable of distinguishing separate contributions of iron in tetrahedral and octahedral positions in the magnetite structure, thus allowing the study of the local structure.^5^ The catalytic performance of these samples was evaluated under industrially relevant conditions for 4 days and at a total pressure of 25 bar.

## Experimental Section

### Catalyst Preparation

Catalysts were prepared via a single-step coprecipitation/calcination procedure adapted from ref (24). Appropriate amounts of the nitrate salts of Fe^3+^, Cr^3+^, Al^3+^, Ga^3+^, In^3+^, Mn^2+^, Zn^2+^, and Cu^2+^ and a Nb^5+^ salt (ammonium niobate oxalate hydrate) were dissolved in deionized water and heated at 60 °C. A NaOH solution was added at this temperature under vigorous stirring until the pH reached 10, followed by ageing the resulting slurry at 60 °C under vigorous stirring for 1 h. The precipitates were filtered, washed, and dried at 150 °C for 3 h, followed by calcination at 400 °C for 4 h in static air. The target dopant/iron atomic ratio of 8.4% was chosen to correspond with the Cr doping level in 8 wt % Cr_2_O_3_/α-Fe_2_O_3_. A CuO doping level of 3 wt % CuO was used, typical for a commercial catalyst composition. Freshly calcined catalysts will be referred to as M-HM or MCu-HM, where M is the metal dopant and HM the hematite phase. Some characterization data of the HM, Cr-HM, and CrCu-HM reference catalysts were published elsewhere.^5,6^

### Characterization

X-ray powder diffraction (XRD) patterns were recorded on a PANalytical X’pert pro diffractometer using Cu Kα radiation. HighScore Plus software was used for spectral fitting. Discharged catalysts were stored in an Ar atmosphere before and during measurements. Transmission ^57^Fe Mössbauer spectra were recorded using a ^57^Co (Rh) source with constant-acceleration or sinusoidal velocity spectrometers. Calibration was performed relative to α-Fe at room temperature. The source and the absorbing samples were kept at the same temperature during measurements. Spectral fitting was performed using Mosswinn 4.0 software. Hyperfine magnetic field values fitted with a distribution fit are reported as averages. Fixed values are indicated when applied. Nitrogen physisorption was carried out on a Micromeritics 2420 ASAP instrument. Samples were degassed with nitrogen at 140 °C for at least 1 h prior to analysis. X-ray photoelectron spectroscopy (XPS) was performed on a Thermo Scientific K-α spectrometer using an aluminum anode (Al Kα = 1486.6 eV). The binding energy was calibrated relative to adventitious carbon at a binding energy (BE) of 285 eV and CasaXPS software (version 2.3.19PR1.0) was used for spectral fitting. Samples were placed on a carbon tape and transferred to the spectrometer under vacuum.

### Catalytic Activity Measurements

Catalytic performance testing was conducted in a parallel microreactor setup. The reactor tubes were charged with calcined catalyst precursors diluted with α-Al_2_O_3_. Prior to activity measurements, the reactor tubes were purged with nitrogen. Catalysts were activated in the presence of process gas (37% H_2_, 9% CO, 4% CO_2_, 17% N_2_, 33% H_2_O) and heated at a temperature of 450 °C when the catalysts were thermally aged for 24 h. The temperature was lowered to 360 °C for activity measurements (24 h). The catalysts were then aged once more for 24 h at 450 °C, followed by a final activity measurement at 360 °C (24 h). Continuous gas-phase analysis by an infrared gas analyzer allowed determining the CO conversion. At the end of the activity tests, the reactors were cooled to 250 °C, followed by a switch from the reaction gas mixture to N_2_. Steam addition was switched off after CO was not observed anymore in the effluent stream. Samples were kept under N_2_ after the reaction, before being stored in a glovebox under Ar atmosphere. The used catalysts are named in a similar fashion as the calcined catalysts, with HM (hematite) being replaced by MG for magnetite. Some characterization data of the MG, Cr-MG, and CrCu-MG reference catalysts were published elsewhere.^5,6^

## Results and Discussion

### Characterization of Catalyst Precursors

**Figure 1:**
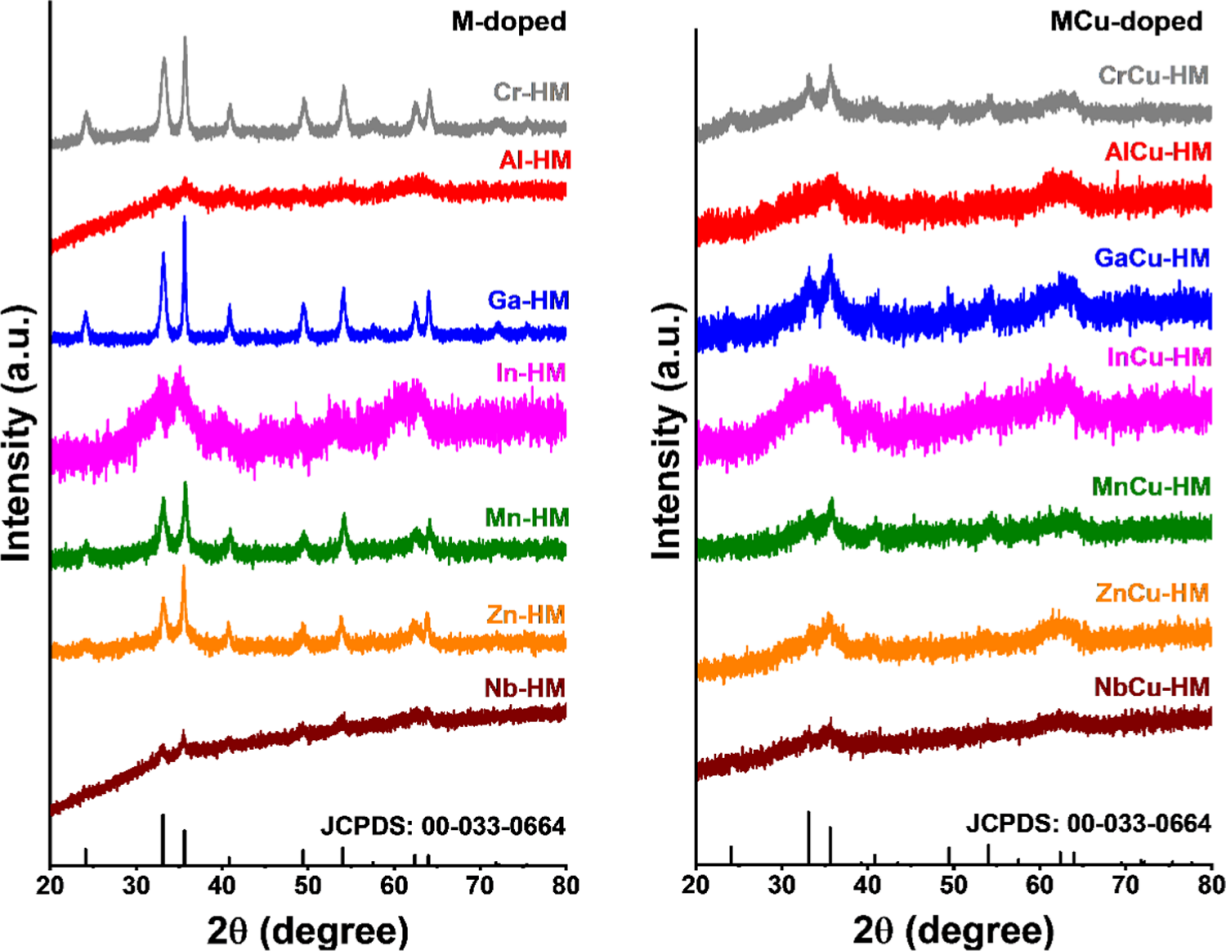
XRD patterns of calcined catalysts: M-doped (left) and MCu-doped (right). JCPDS: 00-033-0664 was used as a hematite reference pattern.

A set of metal-doped iron oxide HTS catalysts was prepared by coprecipitation of metal salts followed by calcination according to a method adapted from Meshkani and Rezaei.^24^ Nondoped hematite (HM) and chromium-doped hematite (Cr-HM) samples are used as reference samples for the discussion of the characterization and catalytic performance results. The physicochemical properties of the calcined catalyst precursors are collected in Table 1 and the XRD patterns in Figure 1. The dopant content of the samples was close to the intended loading of 8.4 mol %. This ratio was chosen on the basis of the optimum chromium loading (8 wt % Cr_2_O_3_ in α-Fe_2_O_3_, 8.4 mol % Cr) typical for a commercial HTS catalyst.^24^ The dopant contents of the gallium- and niobium-doped catalysts were slightly lower than intended.

**Table 1:** Physicochemical Properties of Calcined M-Doped and MCu-Doped Catalysts

sample	dXRD^a^ (nm)	SSA^b^ (m^2^ g^–1^)	*V*_pore_ (cm^3^ g^–1^)	*d*_pore_ (nm)	MO*_x_*^c^ (wt %)	*M* (mol %)	CuO (wt %)	Na_2_O^c^ (wt %)
HM^d^	44	45	0.22	19.9	0	0	0	0.6
Cr-HM^d^	25	110	0.25	9.2	7.5	7.7	0	1.5
CrCu-HM^d^	*	132	0.20	6	8.4	8.7	3.1	0.7
Al-HM	*	140	0.26	7.4	5.3	8.0	0.0	1.0
AlCu-HM	*	168	0.26	6.1	5.2	7.8	3.0	0.6
Ga-HM	41	51	0.23	18	5.8	5.0	0.0	0.5
GaCu-HM	*	133	0.21	6.4	5.6	4.8	3.0	0.4
In-HM	*	124	0.24	7.8	12.7	7.6	0.0	0.8
InCu-HM	*	153	0.26	6.7	12.9	7.8	2.8	0.4
Mn-HM	17	105	0.22	8.4	8.4	8.4	0.0	0.4
MnCu-HM	*	153	0.27	7.1	8.3	8.3	2.9	0.2
Zn-HM	23	93	0.22	9.6	8.3	8.1	0.0	0.5
ZnCu-HM	*	137	0.21	6.1	8.6	8.4	2.9	0.1
Nb-HM	*	140	0.25	7.3	3.3	5.4	0.0	1.6
NbCu-HM	*	155	0.26	6.7	3.4	5.6	3.1	1.2

a Calculated with the Scherrer equation from the α-Fe_2_O_3_ (110) reflection.

b Specific surface area (SSA), pore volume (*V*_pore_), and pore diameter (*d*_pore_) determined by the Brunauer–Emmett–Teller (BET) method.

c Obtained by XRF analysis.

d From ref (6).

XRD patterns of all calcined samples (Figure 1) show reflections that can be attributed to hematite. Chromium is known to prevent thermal agglomeration during calcination of the oxide/hydroxide precursor. The XRD patterns of MCu-HM catalysts are significantly broadened compared to those of M-HM and HM catalysts, which suggests the presence of smaller hematite particles in the Cu-doped samples. However, the reflections observed can also be attributed to the presence of ferrihydrite. Smaller HM particles were observed previously in calcined chromium–copper-codoped HTS catalysts compared to a chromium-doped catalyst.^6^ No diffraction lines of dopant oxide phases were observed in the XRD patterns. This may be taken as an indication that most of the dopants end up in the hematite structure, although we cannot exclude the presence of segregated dopant oxide particles with small crystallite size (<3–4 nm) or amorphous nature.^12^ Similarly, the presence of noncrystalline or weakly crystalline Fe-oxide phases cannot be excluded based on the XRD results.

**Figure 2:**
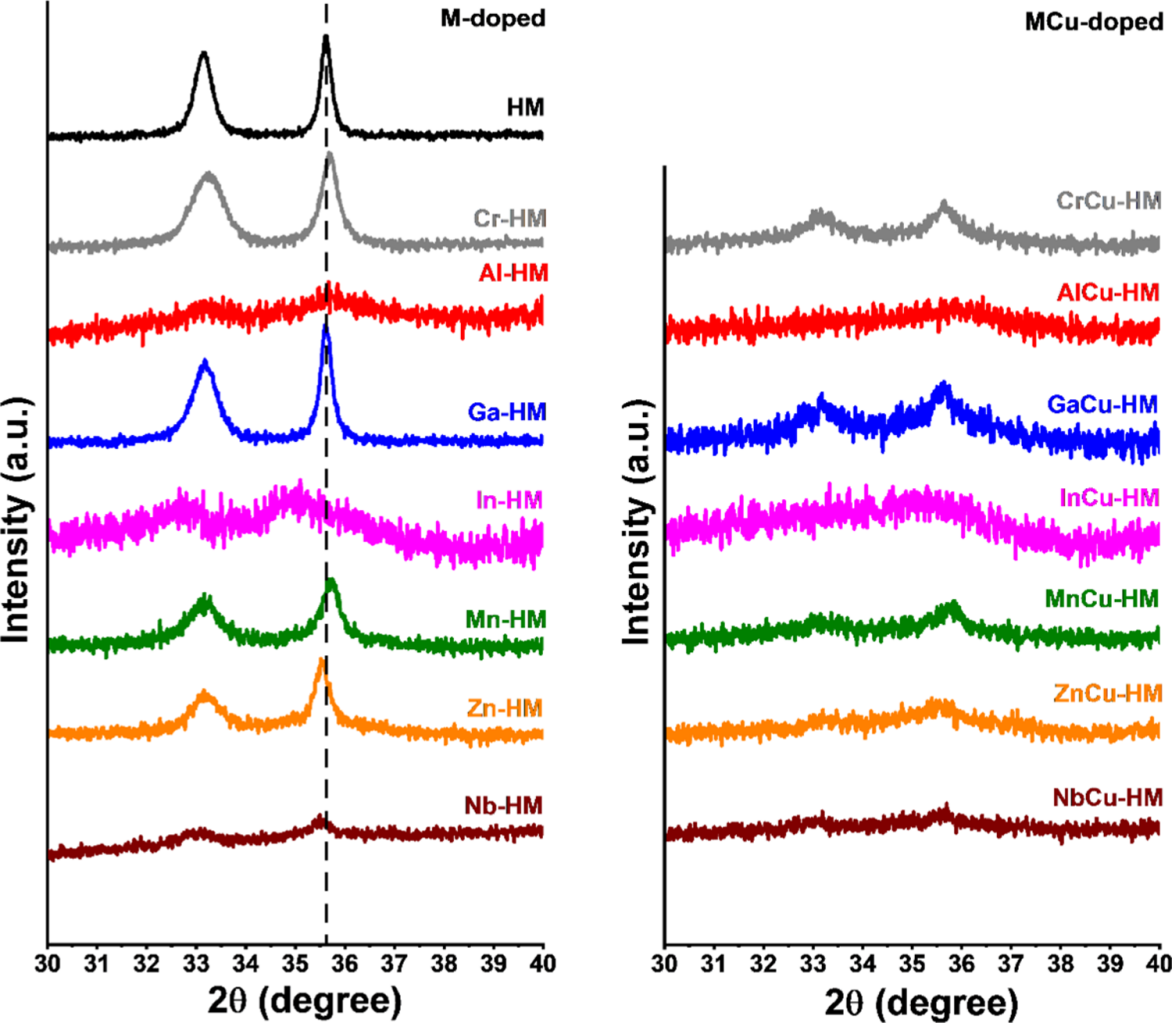
XRD patterns of freshly calcined catalysts in the 2θ range of the (110) reflection of hematite of M-HM catalysts (left) and MCu-HM catalysts (right).

A magnification of the 2θ range corresponding to the (110) reflection of hematite is provided in Figure 2. In Cr-HM, Al-HM, and Mn-HM samples, the (110) reflection is shifted to higher 2θ values compared to the HM reference, indicating that the unit cell is smaller upon doping. This is expected^12^ for the incorporation of dopants (62 pm, Cr^3+^ (oct.); 54 pm, Al^3+^ (oct.)) with smaller ionic radii than iron (65 pm, Fe^3+^ (oct.)). The shift to a higher 2θ value in the Mn-doped catalyst can be explained by oxidation of the initially present Mn^2+^ (oct.) (83 pm) to Mn^4+^ (oct.) (53 pm) upon calcination in air. The (110) reflection of the In-HM, Zn-HM, and Nb-HM catalysts shifted to lower 2θ values, which implies increased unit cell dimensions upon doping. This is in line with the larger ionic radii of indium and zinc (80 pm, In^3+^; 74 pm, Zn^2+^) than Fe^3+^. The shifted 2θ value of the Nb-HM catalyst cannot be explained by the octahedral ionic radius of Nb^5+^ (64 pm), which is similar to that of Fe^3+^. No shift was observed for the Ga-HM sample, which may be due to its very similar ionic radius (62 pm, Ga^3+^ (oct.)) to Fe^3+^ or a low substitution level.

With the exception of Ga-HM, Mn-HM, and Zn-HM catalysts, all samples containing alternative dopants displayed a higher surface area with respect to the HM and Cr-HM references. This suggests an increased resistance against agglomeration of the precursor oxides during the calcination step.

**Figure 3:**
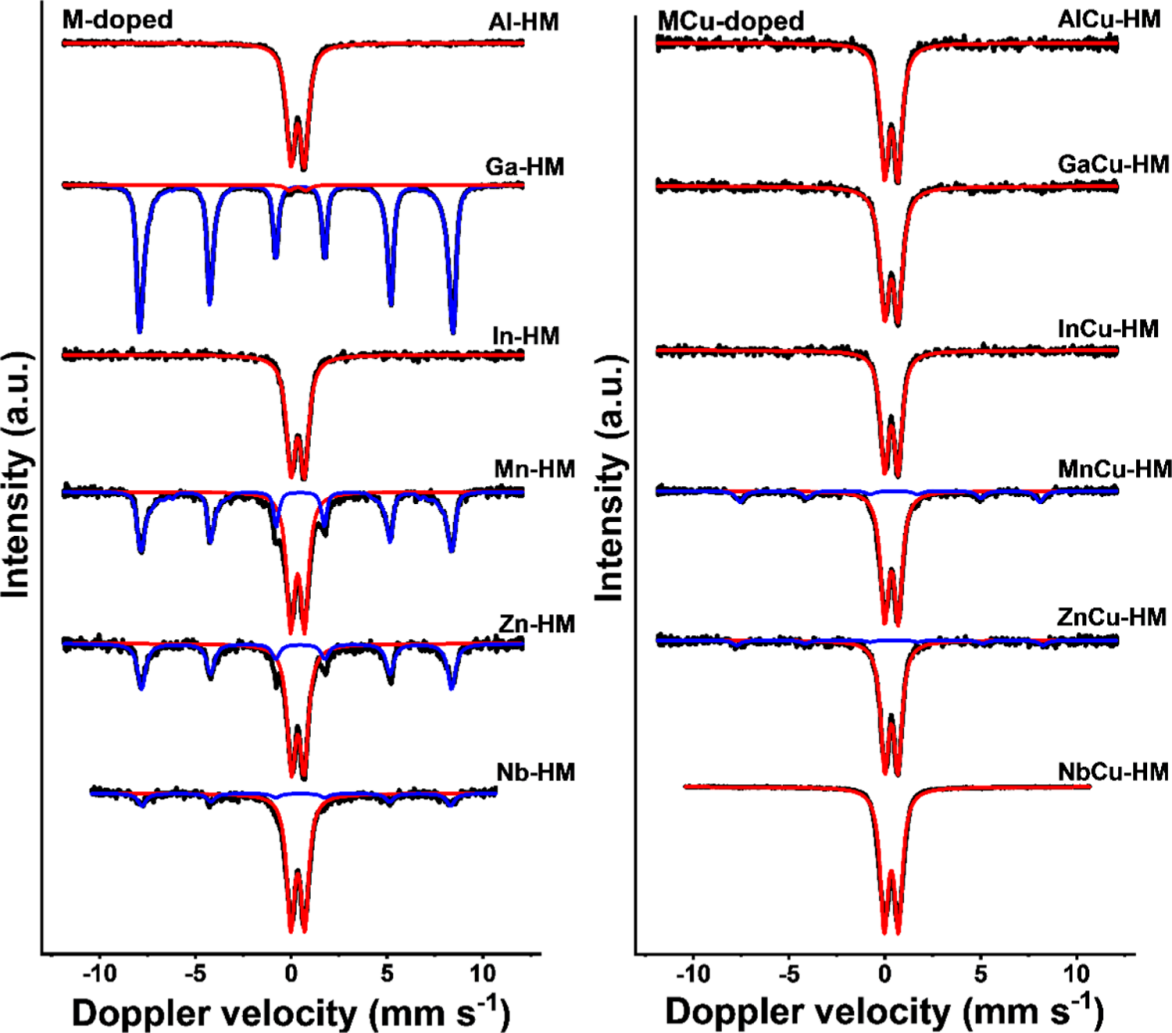
Room-temperature Mössbauer spectra of M-doped (left) and MCu-doped (right) HTS catalysts.

To further investigate the highly dispersed or amorphous iron oxide phases present in these samples, temperature-dependent Mössbauer spectroscopy measurements were performed. Room-temperature Mössbauer spectra are provided in Figure 3. A magnetically split sextet with IS (isomer shift) values of ∼0.37 mm s^–1^, QS (quadrupole splitting) values of approximately −0.21 mm s^–1^, and a hyperfine magnetic field between 50.5 and 48.5 T (Table 2) was observed for Ga-HM, Mn-HM, Zn-HM, Nb-HM, MnCu-HM, and ZnCu-HM catalysts. These hyperfine parameters point to the presence of hematite, in line with XRD patterns.^20^ Apart from the magnetically split hematite phase, a superparamagnetic (SPM) phase with an IS of ∼0.34 mm s^–1^ was observed in all catalysts, which points to a phase with small particles with high spin Fe^3+^ in octahedral positions,^25^ such as in hematite or ferrihydrite.^26^ Accurate-phase identification of the SPM phase cannot be obtained from these room-temperature Mössbauer spectra alone.

**Table 2:** Mössbauer Parameters upon Deconvolution of Room-Temperature Spectra of Calcined HTS Catalysts^a^,^b^

sample	IS (mm s^–1^)	QS (mm s^–1^)	*H* (T)	Γ (mm s^–1^)	phase	spectral contribution (%)
HM^c^	0.38	–0.23	50.5^†^	0.23	α-Fe_2_O_3_	100
Cr-HM^c^	0.38	–0.21	48.5^†^	0.25	α-Fe_2_O_3_	100
CrCu-HM^c^	0.34	0.71		0.59	Fe^3+^ SPM	100
Al-HM	0.34	0.70		0.53	Fe^3+^ SPM	100
AlCu-HM	0.34	0.70		0.51	Fe^3+^ SPM	100
Ga-HM	0.37	–0.21	49.9^†^	0.26	α-Fe_2_O_3_	98
	0.34	0.87		0.50*	Fe^3+^ SPM	2
GaCu-HM	0.34	0.71		0.56	Fe^3+^ SPM	100
In-HM	0.34	0.70		0.55	Fe^3+^ SPM	100
InCu-HM	0.34	0.71		0.50*	Fe^3+^ SPM	100
Mn-HM	0.37	–0.20	48.8^†^	0.26	α-Fe_2_O_3_	50
	0.34	0.73		0.50*	Fe^3+^ SPM	50
MnCu-HM	0.37*	–0.21*	48.6	0.50*	α-Fe_2_O_3_	16
	0.34	0.71		0.50*	Fe^3+^ SPM	84
Zn-HM	0.38	–0.20	50.3	0.29	α-Fe_2_O_3_	42
	0.35	0.66		0.52	Fe^3+^ SPM	58
ZnCu-HM	0.37*	–0.21*	49.7	0.50	α-Fe_2_O_3_	8
	0.34	0.69		0.50*	Fe^3+^ SPM	92
Nb-HM	0.37	–0.21*	50.1	0.50*	α-Fe_2_O_3_	17
	0.34	0.73		0.50*	Fe^3+^ SPM	83
NbCu-HM	0.34	0.74		0.50*	Fe^3+^ SPM	100

a Fixed values are marked with * and average values with †.

b Experimental uncertainties: IS ± 0.01 mm s^–1^, QS ± 0.01 mm s^–1^, line width: Γ ± 0.01 mm s^–1^, hyperfine magnetic field: *H* ± 0.1 T, spectral contribution: ±3%.

c From ref (6).

**Figure 4:**
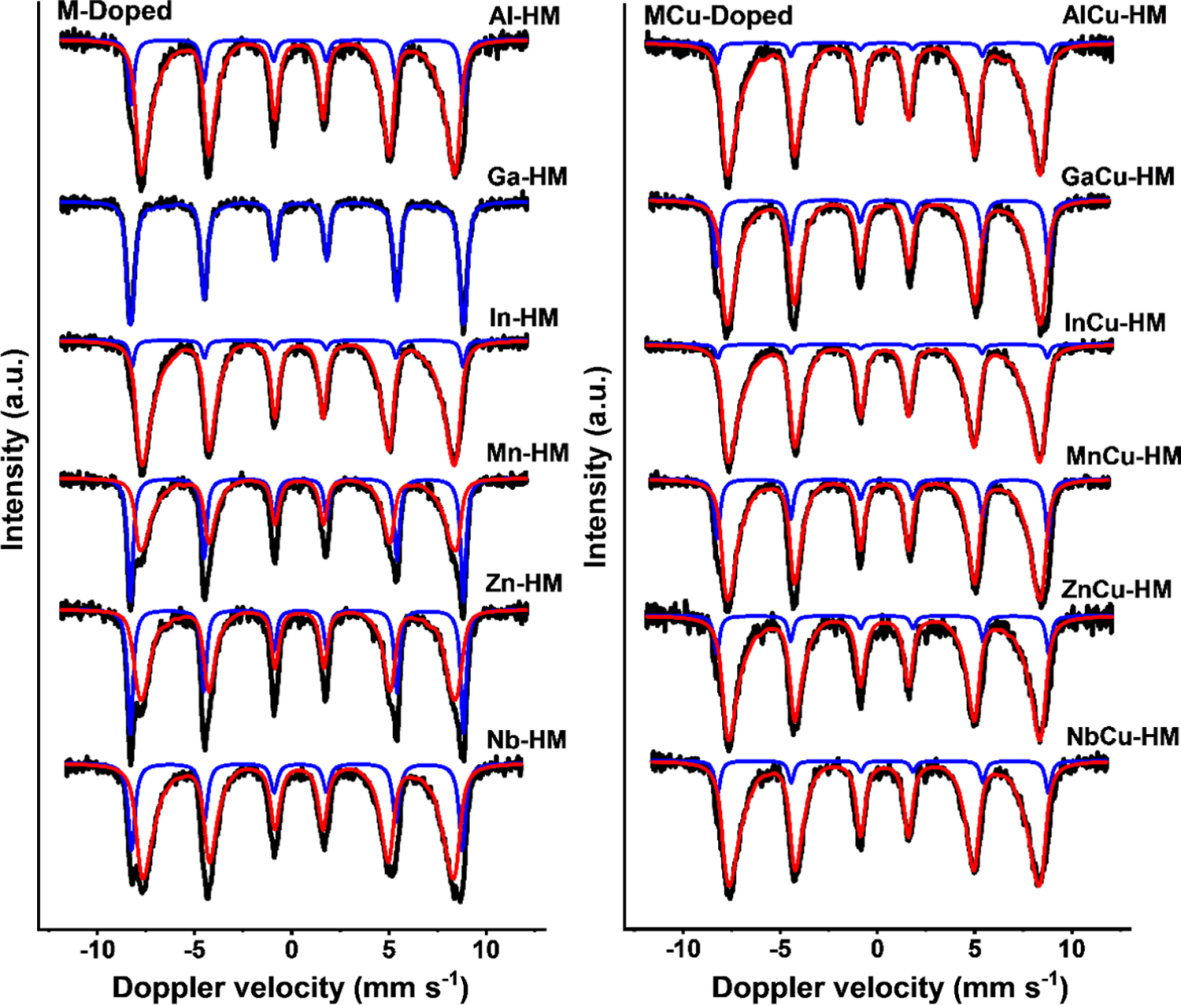
Mössbauer spectra of M-doped (left) and MCu-doped (right) catalysts recorded at −269 °C.

Mössbauer spectra recorded at −269 °C provide deeper insight into the SPM phases (Figure 4). Two spectral contributions are observed for all catalysts except for HM, Cr-HM, and Ga-HM catalysts where only one sextet was observed. The results of the deconvolution of these spectra are given in Table 3. The Mössbauer parameters of the sextets (IS ∼ 0.36 mm s^–1^, hyperfine magnetic fields > 52 T) observed in all catalysts confirm the presence of a hematite phase. The second magnetically split sextet with typical IS values of ∼0.35 mm s^–1^ and a hyperfine magnetic field below 49 T are characteristic of ferrihydrite (Fe_5_HO_8_·4H_2_O).^26^ The data show that the amount of hematite in the catalyst precursor decreased when copper was codoped, indicating that incorporation of copper in the iron oxide precursor prevented hematite formation during the calcination step. In the HM, Cr-HM, and Ga-HM catalysts, only a hematite phase was observed, while Al-HM, In-HM, Mn-HM, Zn-HM, and Nb-HM catalysts contained both hematite and ferrihydrite. This shows that all alternative dopants except for gallium prevent hematite formation under these conditions, suggesting their incorporation into the ferrihydrite structure. The Mössbauer spectroscopy data indicate no significant gallium incorporation into the structure, in line with the large average crystallite size and low SSA for the Ga-doped material (Table 1). Mössbauer spectroscopy allows for quantification of the ferrihydrite content, which is not possible with XRD analysis due to the overlap of ferrihydrite and hematite signals. In addition, the presence of significant levels of a ferrihydrite phase shows that the incorporation of dopants cannot be deduced from XRD analysis, using the shift of the hematite (110) reflection discussed above, as XRD analysis is not able to discriminate between ferrihydrite and weakly crystalline hematite phases.

**Table 3:** Mössbauer Parameters of Calcined Catalysts of Spectra Recorded at −269 °C^a^,^b^

sample	IS (mm s^–1^)	QS (mm s^–1^)	*H* (T)	Γ (mm s^–1^)	phase	spectral contribution (%)
Fe-HM^c^	0.35	0.40	53.6^†^	0.28	α-Fe_2_O_3_	100
Cr-HM^c^	0.36	–0.21	52.7^†^	0.26	α-Fe_2_O_3_	100
CrCu-HM^c^	0.36	–0.21	53.4	0.29	α-Fe_2_O_3_	14
	0.36	–0.04	48.9	0.48	Fe_5_HO_8_·4H_2_O	86
Al-HM	0.35	–0.14	53.1	0.34	α-Fe_2_O_3_	17
	0.35	–0.05	48.3^†^	0.42	Fe_5_HO_8_·4H_2_O	83
AlCu-HM	0.37*	–0.21*	52.8	0.31	α-Fe_2_O_3_	5
	0.35	–0.05	48.2^†^	0.46	Fe_5_HO_8_·4H_2_O	95
Ga-HM	0.36	–0.18	52.5^†^	0.30	α-Fe_2_O_3_	100
GaCu-HM	0.37*	–0.21*	53.0	0.35	α-Fe_2_O_3_	19
	0.35	–0.05	48.7^†^	0.38	Fe_5_HO_8_·4H_2_O	81
In-HM	0.37	–0.14	52.9	0.30	α-Fe_2_O_3_	7
	0.35	–0.04	48.2^†^	0.42	Fe_5_HO_8_·4H_2_O	93
InCu-HM	0.37*	–0.21*	52.7	0.30*	α-Fe_2_O_3_	4
	0.35	–0.03	48.0^†^	0.45	Fe_5_HO_8_·4H_2_O	96
Mn-HM	0.36	–0.19	53.2	0.33	α-Fe_2_O_3_	40
	0.36	–0.07	48.4^†^	0.40	Fe_5_HO_8_·4H_2_O	60
MnCu-HM	0.37	–0.21*	52.9	0.34	α-Fe_2_O_3_	17
	0.35	–0.05	48.4^†^	0.44	Fe_5_HO_8_·4H_2_O	83
Zn-HM	0.36	–0.18	53.2	0.33	α-Fe_2_O_3_	35
	0.36	–0.08	48.8^†^	0.40	Fe_5_HO_8_·4H_2_O	65
ZnCu-HM	0.38	–0.21*	53.0	0.33	α-Fe_2_O_3_	10
	0.36	–0.02	48.0^†^	0.49	Fe_5_HO_8_·4H_2_O	90
Nb-HM	0.35	–0.17	52.8	0.40	α-Fe_2_O_3_	25
	0.35	–0.05	48.0^†^	0.51	Fe_5_HO_8_·4H_2_O	75
NbCu-HM	0.39	–0.21*	52.9	0.34	α-Fe_2_O_3_	9
	0.35	–0.03	47.9^†^	0.48	Fe_5_HO_8_·4H_2_O	91

a Fixed values are marked with * and average values with †.

b Experimental uncertainties: Isomer shift: IS ± 0.01 mm s^–1^, quadrupole splitting: QS ± 0.01 mm s^–1^, line width: Γ ± 0.01 mm s^–1^, hyperfine magnetic field: *H* ± 0.1 T, spectral contribution: ±3%.

c From ref (6).

### Catalytic Activity Testing

**Figure 5:**
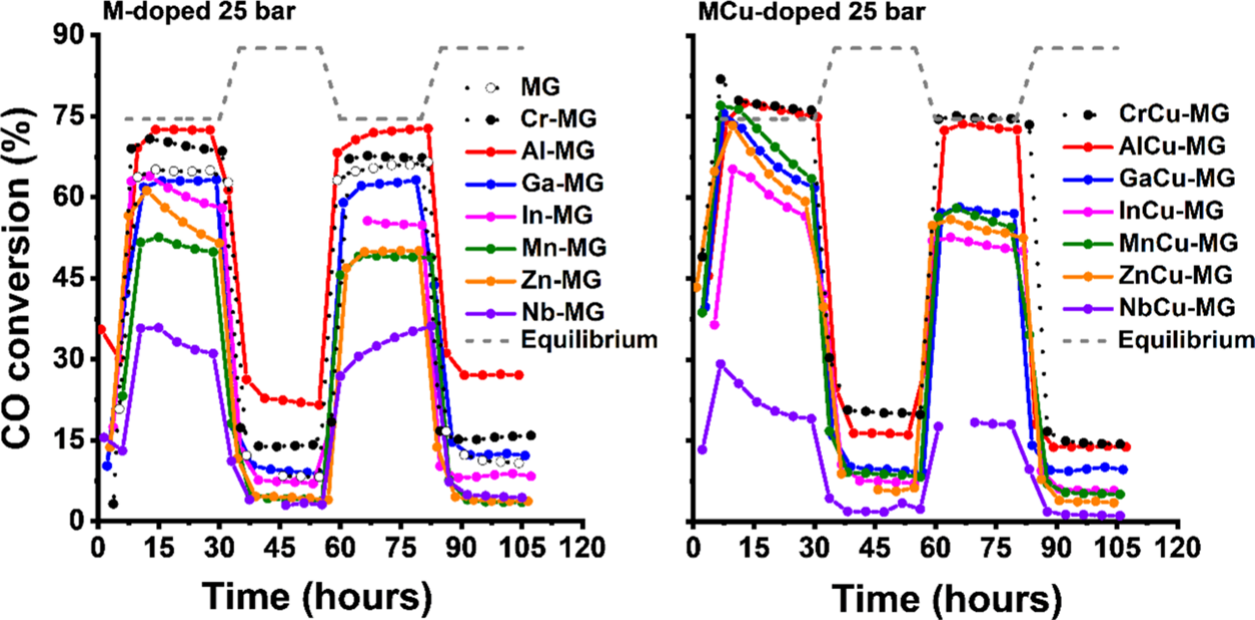
CO conversion under HTS conditions at 25 bar with time on stream of M-doped catalysts (left) and MCu-doped catalysts (right). The temperature was varied between 450 and 360 °C with 24 h intervals. Data of MG, Cr-MG, and CrCu-MG was reproduced from ref (6). The initial CO conversion beyond the equilibrium conversion of the MCu-doped samples can be attributed to magnetite formation.

The catalytic performance of the calcined catalysts was evaluated under close-to-industrial HTS conditions. Figure 5 shows the CO conversion of the M-HM and MCu-HM catalysts at 25 bar as a function of time on stream. An accelerated ageing protocol was carried out for 4 days involving activation, ageing, and activity testing of the catalysts at a pressure of 25 bar using a reaction gas mixture typical for HTS of the effluent of a steam methane reformer. Initial activation was done at 450 °C for 24 h, where the slightly higher than equilibrium conversion for the MCu-doped samples is likely due to the reduction of the precursor Fe-oxides to magnetite. Catalytic performance of various samples was then evaluated at 360 °C for 24 h, followed by an ageing step at 450 °C for 24 h and another catalytic activity test at 360 °C for 24 h. As it has been well established that the iron oxide precursor phase reduces into the active magnetite phase during the initial phases of the HTS reaction, we will denote the used catalysts as M-MG and MCu-MG. The comparison of the copper-codoped catalysts is most meaningful as the presence of copper promotes the activation of the catalyst by enhancing the partial reduction of the iron oxide precursor phases. While the Al-MG catalyst shows a higher activity than the Cr-MG sample, the CO conversion levels of their copper-promoted counterparts are very similar, especially after the high-temperature ageing step. This shows that aluminum doping of Fe-based WGS catalysts can lead to a similar activity and stability as chromium doping under industrial HTS conditions. Earlier, such promising effect of aluminum doping was reported at atmospheric pressure and for relatively short reaction times.^15^ The other catalysts show an appreciably lower CO conversion than these two, with the activity decreasing in the order gallium > indium > manganese > zinc > niobium. As one may expect similar chemical behavior of Ga and Al, it could be that the lower performance of Ga-MG and GaCu-MG catalysts is due to the low gallium content and/or low incorporation level in the fresh catalyst. The HTS activity upon doping with group 13 elements decreases in the order Al > Ga > In.

### Characterization of Used Catalysts

**Figure 6:**
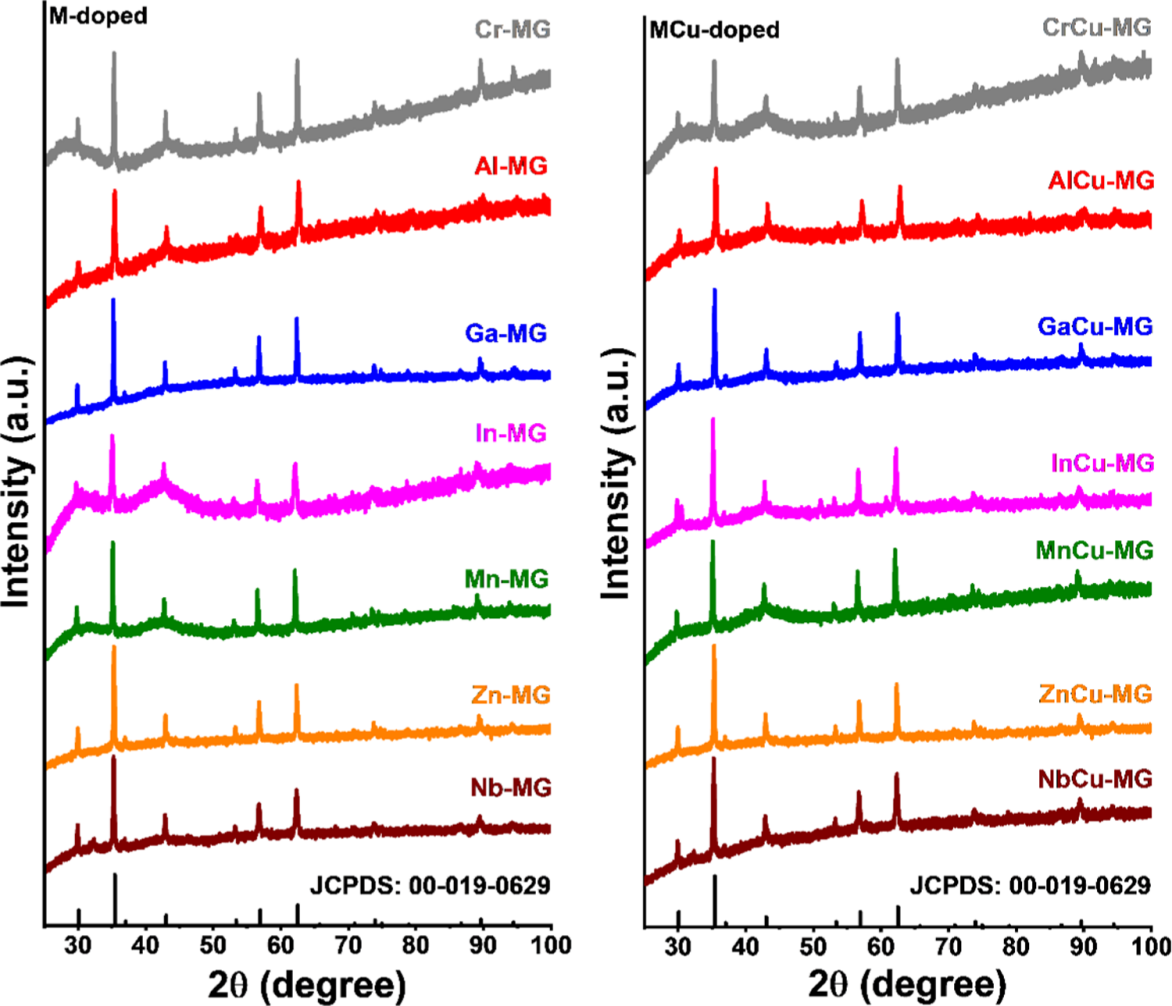
XRD patterns of used catalysts (M-doped, left; MCu-doped, right) after 4 days at 25 bar. JCPDS: 00-019-0629 represents magnetite.

The XRD patterns of the discharged catalysts in Figure 6 contain reflections that can be attributed to magnetite or maghemite (γ-Fe_2_O_3_) phases. Despite their very similar diffraction patterns, we assign them to magnetite, as it is well accepted that exposure of Fe^3+^-oxides to WGS conditions results in this phase.^7^ The indium- and niobium-containing samples exhibited additional reflections at 2θ = ∼30.5° and 2θ = ∼32.3°, respectively (Figures 6 and S1). The 2θ reflection at 30.5° for the former is the (222) reflection of In_2_O_3_^27^ and its formation is likely due to the relatively large ionic radius of indium ions (In^3+^ (oct.), 80 pm) compared to ferric ions (Fe^3+^ (oct.), 65 pm). The reflection at 2θ = ∼32.3° for the niobium-containing samples can be linked to the formation of FeNbO_4_^28^ and FeNb_2_O_6_.^29^ Similar to the calcined catalysts, the unit cell of magnetite is contracted for Cr-MG and Al-MG catalysts and expanded for In-MG, Zn-MG, and Nb-MG ones. Interestingly, the lattice of the Mn-MG catalyst is expanded, while a lattice contraction was observed in the Mn-HM catalyst. No significant differences were observed between the XRD patterns of the M-MG and MCu-MG catalysts, with the exception of the Ga-MG and GaCu-MG catalysts where lattice expansion and contraction occur, respectively. Because of the different sizes of the various dopants in tetrahedral and octahedral positions,^30^ it is difficult to correlate the shift in 2θ values to the degree of dopant incorporation.

Crystallite sizes of the used catalysts were determined by XRD line broadening analysis (Table 4). The differences in crystallite size among the copper-doped catalysts are relatively small, with the size of 30 nm of the AlCu-MG standing out. Among the catalysts not containing copper, Al-MG, In-MG, and Nb-MG contain the smallest magnetite crystallites. Clearly, variations in the crystallite sizes cannot account for the differences in the catalytic performance. Therefore, the incorporation of dopants in magnetite was investigated in more detail and an attempt was made to correlate this information to the activity data.

**Table 4:** Particle Size Determined from XRD (*d*_XRD_) of Used Catalysts after 4 Days at 25 Bar

dopant	*d*_XRD_ (nm)^a^	
	M-MG	MCu-MG
−	74	−
Al	35	30
Ga	99	54
In	36	44
Mn	76	57
Zn	83	47
Nb	34	48
Cr	64^b^	43^b^

a Calculated with the Scherrer equation from the magnetite (311) reflection.

b From ref (6).

**Figure 7:**
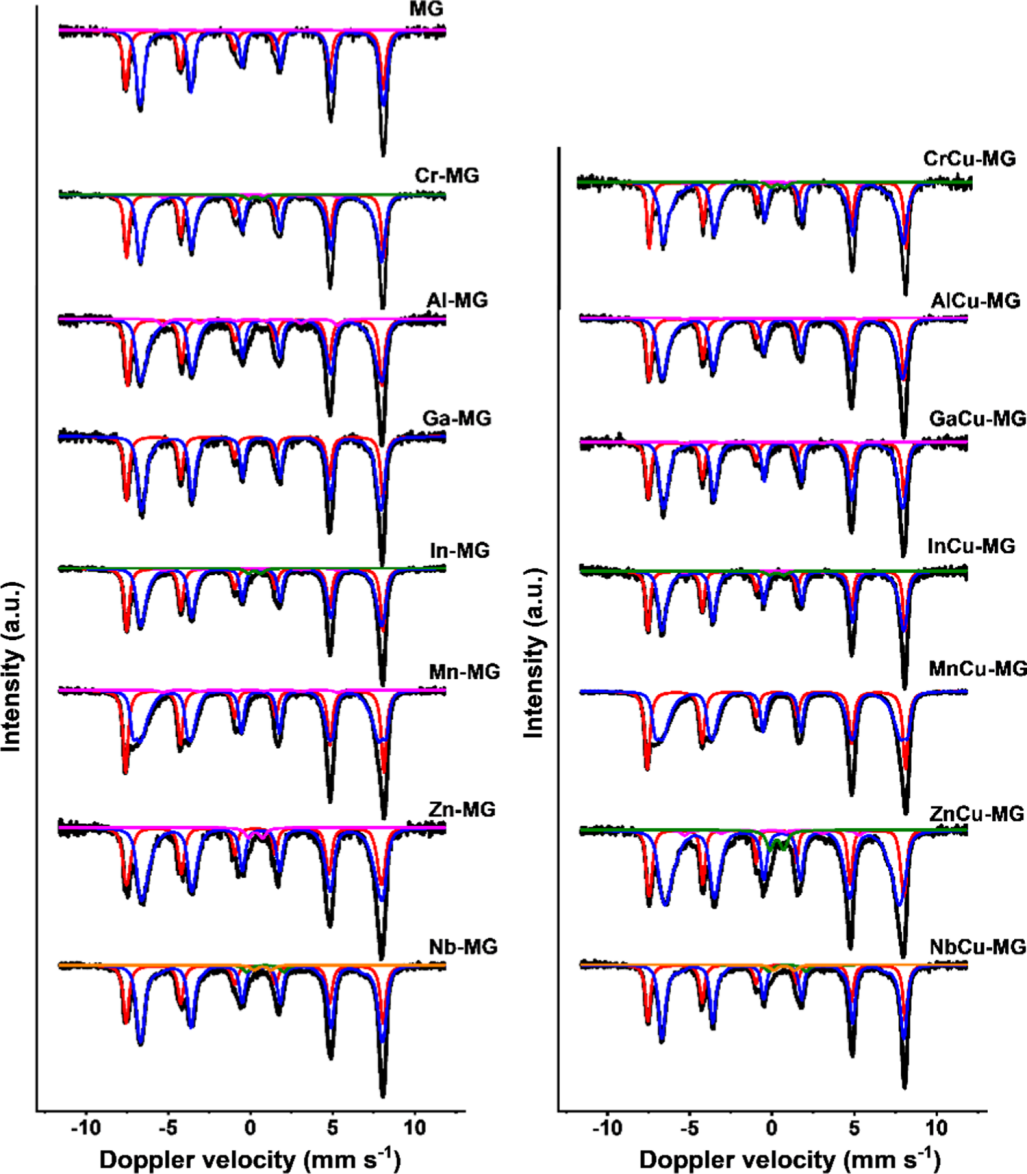
Room-temperature Mössbauer spectra of discharged catalysts after 4 days at 25 bar under HTS conditions.

Mössbauer spectra of the discharged catalysts used in the activity tests for 4 days at 25 bar are shown in Figure 7. The hyperfine parameters obtained after deconvolution are collected in Table 5. A magnetite phase was observed for all used catalysts irrespective of the dopant. The active magnetite catalyst has an inverse spinel structure (AB_2_O_4_) with Fe^3+^ in the tetrahedral A-sites and Fe^3+^/Fe^2+^ in the octahedral B-sites in an equimolar ratio.^31^ The magnetite HTS catalyst follows a regenerative redox mechanism for the WGS reaction where Fe^2+^ is oxidized to Fe^3+^ by H_2_O and subsequently reduced back to Fe^2+^ by CO in the Fe^3+^/Fe^2+^ redox couple.^1^ The room-temperature Mössbauer spectrum of magnetite can be deconvoluted into separate contributions from tetrahedral and octahedral Fe ions.^5^ The separate tetrahedral and octahedral contributions allow us to study the effects of the various dopants on the tetrahedral and octahedral sites in detail together with dopant effects on the Fe^3+^/Fe^2+^ redox couple in the octahedral sites.

**Table 5:** Mössbauer Parameters of Discharged Catalysts after Exposure to HTS Conditions for 4 Days at 25 Bar^a^,^b^

sample	IS (mm s^–1^)	QS (mm s^–1^)	*H* (T)	Γ (mm s^–1^)	phase	spectral contribution (%)
MG^c^	0.26	–0.03	48.7	0.38	Fe_3_O_4_(tet)	37
	0.68	–0.03	45.7^†^	0.32	Fe_3_O_4_(oct)	62
	0.00*	0.00*	33.0*	0.50*	α-Fe	1
Cr-MG^c^	0.28	0.00	48.6	0.34	Fe_3_O_4_(tet)	35
	0.64	–0.01	44.6^†^	0.32	Fe_3_O_4_(oct)	61
	0.00*	0.00*	33.0*	0.50*	α-Fe	2
	0.30*	0.86		0.50*	Fe^3+^ SPM	2
CrCu-MG^c^	0.29	0.00	48.4	0.32	Fe_3_O_4_(tet)	34
	0.64	–0.03	44.2^†^	0.30	Fe_3_O_4_(oct)	62
	0.00*	0.00*	33.0*	0.50*	α-Fe	2
	0.30*	0.76		0.50*	Fe^3+^ SPM	2
Al-MG	0.28	–0.02	48.0	0.37	Fe_3_O_4_(tet)	36
	0.66	–0.02	44.5	0.35	Fe_3_O_4_(oct)	59
	0.00*	0.00*	33.0*	0.50*	α-Fe	5
AlCu-MG	0.28	–0.01	48.1	0.37	Fe_3_O_4_(tet)	36
	0.64	–0.02	44.5^†^	0.35	Fe_3_O_4_(oct)	63
	0.00*	0.00*	33.0*	0.50*	α-Fe	1
Ga-MG	0.27	–0.01	48.4	0.34	Fe_3_O_4_(tet)	35
	0.66	0.00	44.6^†^	0.30	Fe_3_O_4_(oct)	65
GaCu-MG	0.27	–0.02	48.3	0.35	Fe_3_O_4_(tet)	34
	0.67	0.01	44.6^†^	0.29	Fe_3_O_4_(oct)	64
	0.00*	0.00*	33.0*	0.50*	α-Fe	2
In-MG	0.28	–0.02	48.4	0.35	Fe_3_O_4_(tet)	38
	0.66	0.00	44.9^†^	0.32	Fe_3_O_4_(oct)	58
	0.00*	0.00*	33.0*	0.50*	α-Fe	2
	0.25	0.77		0.50*	Fe^3+^ SPM	2
InCu-MG	0.28	–0.02	48.5	0.30	Fe_3_O_4_(tet)	35
	0.66	0.00	44.9^†^	0.30	Fe_3_O_4_(oct)	61
	0.00*	0.00*	33.0*	0.50*	α-Fe	2
	0.29	0.87		0.50*	Fe^3+^ SPM	2
Mn-MG	0.28	–0.03	48.8	0.36	Fe_3_O_4_(tet)	39
	0.58	–0.04	45.1^†^	0.31	Fe_3_O_4_(oct)	59
	0.00*	0.00*	33.0*	0.50*	α-Fe	2
MnCu-MG	0.28	–0.03	48.7	0.36	Fe_3_O_4_(tet)	39
	0.59	–0.04	45.0^†^	0.32	Fe_3_O_4_(oct)	61
Zn-MG	0.25	–0.06	48.0	0.33	Fe_3_O_4_(tet)	34
	0.66	0.04	44.4^†^	0.38	Fe_3_O_4_(oct)	63
	0.31	0.86		0.50*	Fe^3+^ SPM	3
ZnCu-MG	0.27	–0.01	48.0	0.24	Fe_3_O_4_(tet)	26
	0.62	0.00	43.4^†^	0.39	Fe_3_O_4_(oct)	66
	0.00*	0.00*	33.0*	0.50*	α-Fe	3
	0.30*	0.84		0.50*	Fe^3+^ SPM	5
Nb-MG	0.27	–0.03	48.6	0.40	Fe_3_O_4_(tet)	33
	0.66	0.02	45.1^†^	0.36	Fe_3_O_4_(oct)	62
	0.90	2.29		0.50*	FeNb_2_O_6_ (I)	3
	0.75*	1.06		0.50*	FeNb_2_O_6_ (II)	2
NbCu-MG	0.28	0.00	48.6	0.34	Fe_3_O_4_(tet)	33
	0.65	0.00	44.9^†^	0.31	Fe_3_O_4_(oct)	61
	0.94	2.24		0.50*	FeNb_2_O_6_ (I)	3
	0.75*	1.29		0.50*	FeNb_2_O_6_ (II)	3

a Fixed values are marked with * and average values of distribution fits with †.

b Experimental uncertainties: IS ± 0.01 mm s^–1^, QS ± 0.01 mm s^–1^, line width: Γ ± 0.01 mm s^–1^, hyperfine magnetic field: *H* ± 0.1 T, spectral contribution: ± 3%.

c From ref (6).

Deconvolution of the Mössbauer spectra of the Al-MG and AlCu-MG catalysts yields hyperfine magnetic field values of ∼48.0 T for the tetrahedral and ∼44.5 T for the octahedral sites (Table 5 and Figure 7). These values are lower than those of the reference MG catalyst (48.7 and 45.7 T, respectively, Table 5). A decrease of the hyperfine magnetic field from the bulk value of magnetite can be due to a decrease in the particle size or the incorporation of another metal ion in the structure.^5^ Quantification of the dopant level is in theory possible using a set of samples with different dopant levels. However, such approach is hampered by the influence of particle size on the hyperfine magnetic field. Therefore, we discuss the incorporation levels in a qualitative sense in this work. The average crystallite sizes of 35 nm for Al-MG and 30 nm for AlCu-MG are very similar to the crystallite size of 30 nm for a 12 wt % chromium-doped catalyst recently described by us.^5^ The higher hyperfine magnetic field of tetrahedral sites for this latter sample of 48.4 T is due to the preferential doping of chromium in octahedral sites. Taken together, it can be concluded that the lower tetrahedral hyperfine magnetic field for the Al-MG and AlCu-MG catalysts is due to the incorporation of aluminum in the tetrahedral sites of magnetite. The observed hyperfine magnetic field of ∼44.4 T for the octahedral sites means that aluminum is also incorporated in octahedral sites. As the hyperfine magnetic field values and the crystallite sizes are similar for AlCu-MG and Al-MG catalysts, we conclude that copper is not incorporated in the magnetite phase in the used AlCu-MG catalyst. The same holds for CrCu-MG catalysts.^6^ Aluminum incorporation in both tetrahedral and octahedral positions of magnetite has been reported before. Some studies reported that aluminum can be incorporated in both tetrahedral and octahedral sites^32^ using Mössbauer spectroscopy, whereas others indicated a preference for substitution of octahedral iron by aluminum in magnetite by measuring the magnetic susceptibility.^33^ None of these earlier studies pertained to catalysts used under industrial HTS conditions.

The IS value of 0.66 mm s^–1^ for the octahedral sites in the Al-MG catalyst is within the experimental uncertainty of the bulk value of 0.67 mm s^–1^ in nondoped magnetite.^26^ The latter value is the result of fast electron hopping in the equimolar Fe^3+^/Fe^2+^ redox couple in the octahedral sites, which is faster than the lifetime of the relevant excited state in the Mössbauer measurement, resulting in an IS value representing an average oxidation state of Fe^2.5+^.^34^ As IS values of typical Fe^3+^-oxides are around 0.3 mm s^–1^, a value lower than the bulk magnetite value of 0.67 mm s^–1^ can be explained by a higher than unity Fe^3+^/Fe^2+^ ratio.^5^ The IS value of 0.64 mm s^–1^ for the octahedral sites of the AlCu-MG catalyst indicates that a small fraction of Fe^2+^ in these locations was oxidized. Such a decrease in the IS value upon copper doping was not observed for the CrCu-MG catalyst.

Our hyperfine magnetic field data for the AlCu-MG catalysts shows that doping of aluminum occurs in both tetrahedral and octahedral sites of the active magnetite phase. Clearly, such doping contributes to the improved thermal stability of this phase under HTS conditions, which is comparable to the stability during a 4-day test of the CrCu-MG catalyst where chromium is incorporated exclusively in octahedral positions. This shows that the stabilization of the high surface area of magnetite occurs irrespective of the location of dopant.

The hyperfine magnetic field values for the Ga-MG and GaCu-MG catalysts are ∼48.4 T for the tetrahedral sites and 44.6 T for the octahedral sites (Table 5 and Figure 7). The tetrahedral hyperfine magnetic field of ∼48.4 T is only slightly lower than that of the MG reference (48.7 T), indicating that gallium doping in the tetrahedral position is likely limited. The significantly lower octahedral hyperfine magnetic field value of 44.6 T, compared to 45.7 T in the MG reference, in combination with the relatively large crystallite size observed (Table 4) points to preferential gallium incorporation into octahedral sites. The presence of gallium in the activated catalyst suggests that dopant incorporation can occur during the activation procedure since no evidence of gallium incorporation was found in the catalyst precursor. Earlier, Kohout et al.^35^ and Rećko et al.^36^ suggested preferential occupation of tetrahedral sites of magnetite by gallium using NMR and Mössbauer spectroscopy, respectively, whereas a study by Wang et al.^37^ indicated incorporation of gallium into both tetrahedral and octahedral sites using Mössbauer spectroscopy. These inconclusive findings might be due to the fact that the samples were prepared in different ways. The IS values of 0.66 mm s^–1^ for the octahedral sites in Ga-MG and GaCu-MG are close to the one observed for the MG catalyst, indicating that the Fe^3+^/Fe^2+^ ratio is unaffected by gallium doping.

The In-MG and InCu-MG catalysts showed similar tetrahedral hyperfine magnetic field values of ∼48.5 T as MG (48.7 T); see Table 5. The slightly lower hyperfine magnetic field of 44.9 T for the octahedral sites in comparison to the MG reference value of 45.7 T suggests that a small amount of indium is incorporated in the octahedral sites. Thus, it is likely that the extent of indium doping in the active catalyst is small and a significant amount of indium ends up in a separate In_2_O_3_ phase, as confirmed by XRD analysis (Figure 6). The octahedral IS values of 0.66 mm s^–1^ for the two indium-doped catalysts show that indium doping has no significant effect on the Fe^3+^/Fe^2+^ redox couple. It is therefore likely that the relatively large size of In^3+^ compared to Fe^3+^ results in phase segregation during the activation treatment, although In incorporation in the calcined precursor was confirmed by XRD and Mössbauer spectroscopy. Likely, this additional In_2_O_3_ phase blocks the active magnetite sites at the surface, explaining the much lower activity in comparison to the Cr-MG sample.

In the Mn-MG and MnCu-MG catalysts, hyperfine magnetic field values of ∼48.7 and ∼45.0 T were observed for the tetrahedral and octahedral sites of magnetite, respectively (Table 5). These values are relatively close to those of the reference MG catalyst (48.7 and 45.7 T). Nevertheless, the lower octahedral value for manganese-doped samples points to the incorporation of manganese into the magnetite structure. A substantial decrease in the IS values of the octahedral sites to 0.58 mm s^–1^ compared to the bulk value of 0.67 mm s^–1^ points to an increased Fe^3+^/Fe^2+^ ratio, which can be due to the replacement of octahedral Fe^2+^ for Mn^2+^.^5^ Incorporation of Mn^2+^ for Fe^2+^ into octahedral sites of magnetite was reported before by Sorescu et al.^38^ using Mössbauer spectroscopy, in line with our results. This does not, however, exclude the possibility that some Mn^3+^ ions replace Fe^3+^ ions in octahedral sites as well. These results show that doping M^2+^ ions with an octahedral site preference can lead to a significant distortion of the Fe^3+^/Fe^2+^ redox couple. Although Mn^2+^ incorporation occurs in a similar way as Cr^3+^ incorporation by replacing octahedral Fe ions in magnetite, no improved catalyst activity or stability was observed (Figure 5). This highlights the complex correlation between the dopant incorporation and catalyst performance.

In the Zn-MG catalyst, a hyperfine magnetic field value of 48.0 T (Table 5) was observed for the tetrahedral sites of magnetite. This, in combination with relatively large crystallites (Table 4), indicates the incorporation of zinc into the tetrahedral sites, similar to the aluminum-doped catalysts. A tetrahedral site preference was observed before for zinc-doped magnetite.^39,40^ The hyperfine magnetic field of the octahedral sites decreased in the Zn-MG catalyst (44.4 T), which points to zinc incorporation in octahedral sites as well. Zinc can occupy both the tetrahedral and octahedral sites of magnetite at high zinc content^41^ and it has been pointed out before that the preparation procedure can influence the site preference.^42^ The incorporation of divalent zinc into the tetrahedral sites of magnetite where it replaces trivalent iron, would lead to a charge imbalance. Wen et al.^43^ proposed that the charge imbalance can be resolved by partial oxidation of Fe^2+^ to Fe^3+^ in the octahedral sites. This leads to the (Zn_*x*_^2+^Fe_1–*x*_^3+^)[Fe_1–*x*_^2+^Fe_1+*x*_^3+^]O_4_ structure, where the tetrahedral sites are shown in parentheses and the octahedral sites in brackets. In support of this model are the observations of Mendoza Zélis et al.^40^ of a more ferric-like IS in the octahedral sites of their samples with increasing zinc doping in tetrahedral sites. Walz et al.^44^ suggested a different model where zinc was incorporated into both the tetrahedral and octahedral sites of vacancy(Δ)-doped magnetite (Zn_*x*–*y*_^2+^Fe_1–*x*+*y*_^3+^)[Zn_*y*_^2+^Fe_1–*x*_^2+^Fe_1+*x*–*y*_^3+^]_−Δ_O_4_. They proposed that, at low zinc content, Zn^2+^ ions can migrate to octahedral vacancies, resulting in incorporation into both lattice sites of the spinel. The IS value of 0.66 mm s^–1^ measured for the octahedral sites of our Zn-MG catalyst is similar to that of bulk magnetite (0.67 mm s^–1^). As such, this implies that the Wen model cannot describe the cation arrangement in our Zn-MG catalyst. Instead, the Walz model better describes the observed decrease in the hyperfine magnetic field values of the tetrahedral and octahedral sites for our Zn-MG catalyst (Table 5).

A hyperfine magnetic field value of 48.0 T was observed for the tetrahedral sites of the ZnCu-MG catalyst. The decrease from the bulk magnetite value can again be attributed to the incorporation of zinc in tetrahedral sites. Different from the Zn-MG catalyst, the copper-doped sample also led to a substantial decrease in the hyperfine magnetic field of the octahedral sites to 43.4 T. The lower hyperfine magnetic field in the octahedral sites is accompanied by a more ferric-like IS value of 0.62 mm s^–1^. This could indicate the incorporation of Cu^2+^ into the zinc-doped magnetite structure in place of Fe^2+^. However, copper incorporation in magnetite has not been observed before for other copper-doped magnetite samples. It is therefore more likely that the lower IS value in the octahedral sites can be explained by the Wen model, where partial oxidation in the octahedral sites leads to a lower IS. The lower hyperfine magnetic field is due to the presence of small magnetite particles, which is supported by the substantial SPM contribution for ZnCu-MG (Figure 7 and Table 5). Thus, doping magnetite with divalent ions with a preference for tetrahedral sites can result in a complex structure where the charge imbalance is compensated from the octahedral sites by partial oxidation of Fe^2+^ to Fe^3+^, as described by Wen et al.

In the Mössbauer spectra of the Nb-MG and NbCu-MG catalysts (Figure 7), two SPM doublets and two magnetically split sextets were observed. The two SPM doublets with IS values of 0.75 and ∼0.92 mm s^–1^ and QS values of 1.06–1.29 and 2.24–2.29 mm s^–1^, respectively, are typical for an FeNb_2_O_6_ phase.^45^ Hyperfine magnetic field values of 48.6 and ∼45.0 T were observed for the magnetically split sextets. The value of 48.6 T is close to that of nondoped MG, indicating that no niobium substitution occurred in the tetrahedral sites. The hyperfine magnetic field of ∼45.0 T observed for the octahedral sites was slightly lower than that of the reference MG catalyst, indicating some niobium incorporation in the octahedral sites of magnetite. The relatively low influence of niobium on the hyperfine magnetic field values is most likely due to the segregation into a separate phase. IS values of 0.65–0.66 mm s^–1^ for the octahedral sites indicate that no significant change in the Fe^3+^/Fe^2+^ redox couple occurred upon niobium doping. This might be due to the low doping level but also due to the replacement of an equal amount of Fe^2+^ and Fe^3+^ ions for one Nb^5+^ ion. The formation of a separate FeNb_2_O_6_ phase during activation under industrially relevant HTS conditions and the low catalytic performance mean that niobium is unsuitable to replace chromium in HTS catalysts.

### XPS Analysis

**Figure 8:**
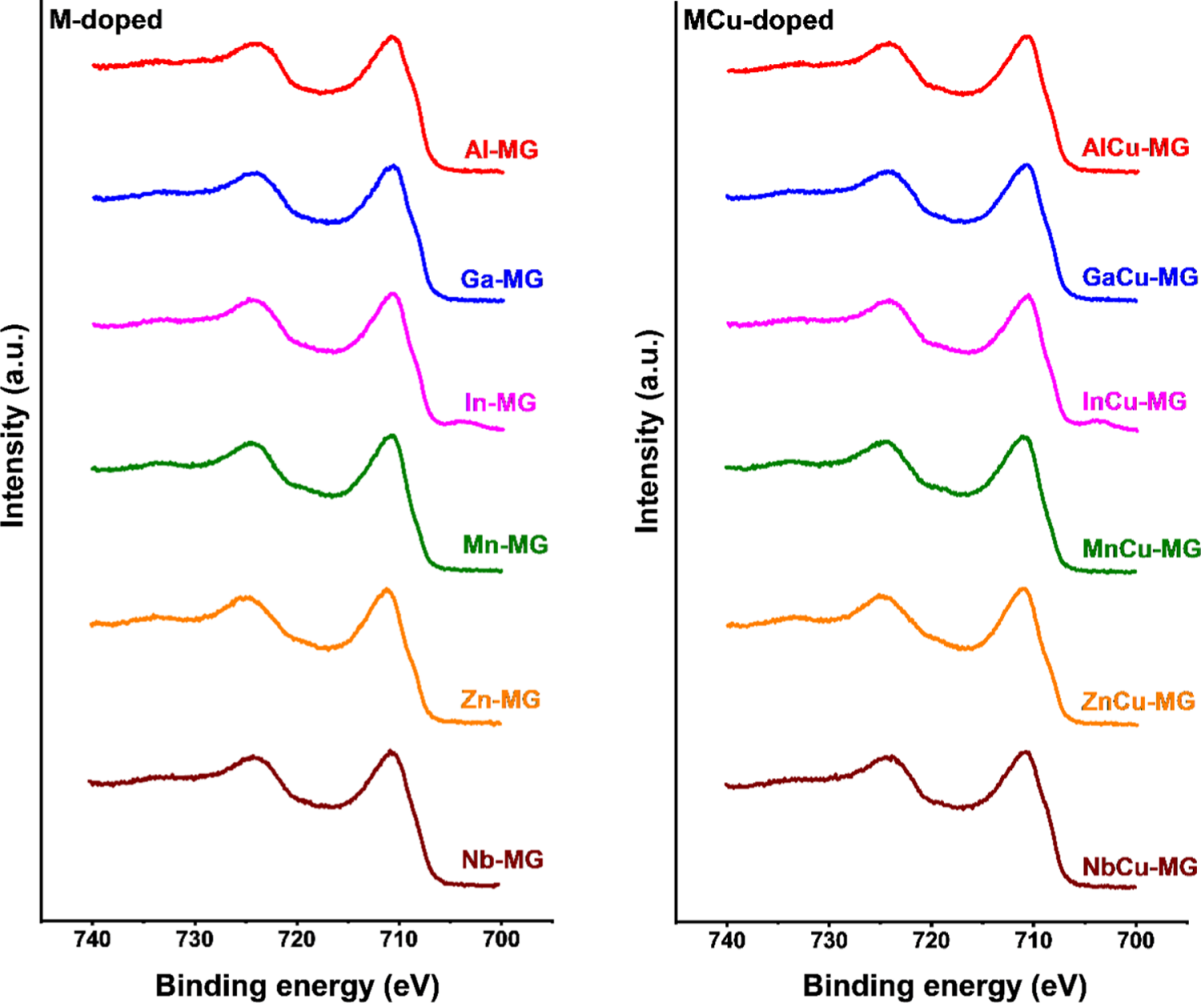
Fe 2p region of discharged M-MG and MCu-MG catalysts after exposure to HTS conditions for 4 days at 25 bar. The small peak observed in the Fe 2p regions of In-MG and InCu-MG spectra at BE = ∼703 eV results from the In 3p peak.

The surface of the catalysts aged under HTS conditions for 4 days at 25 bar was investigated by XPS. The Fe 2p spectra of the M- and MCu-doped catalysts are shown in Figure 8. The Fe^3+^ and Fe^2+^ satellite peaks expected for the corresponding pure Fe_2_O_3_ and Fe_1–*y*_O oxides at binding energies of 719 and 715.5 eV^46^ are not observed in the XPS spectra of the used catalysts. This is typical for materials in which magnetite is the dominant iron oxide phase. It also implies that the surface of the catalysts is very similar to the bulk magnetite structure as shown by XRD and Mössbauer spectroscopy. XPS spectra of valence states of the dopants are provided in the supporting information (Figure S2). All dopants are present in the expected oxidation states.

**Figure 9:**
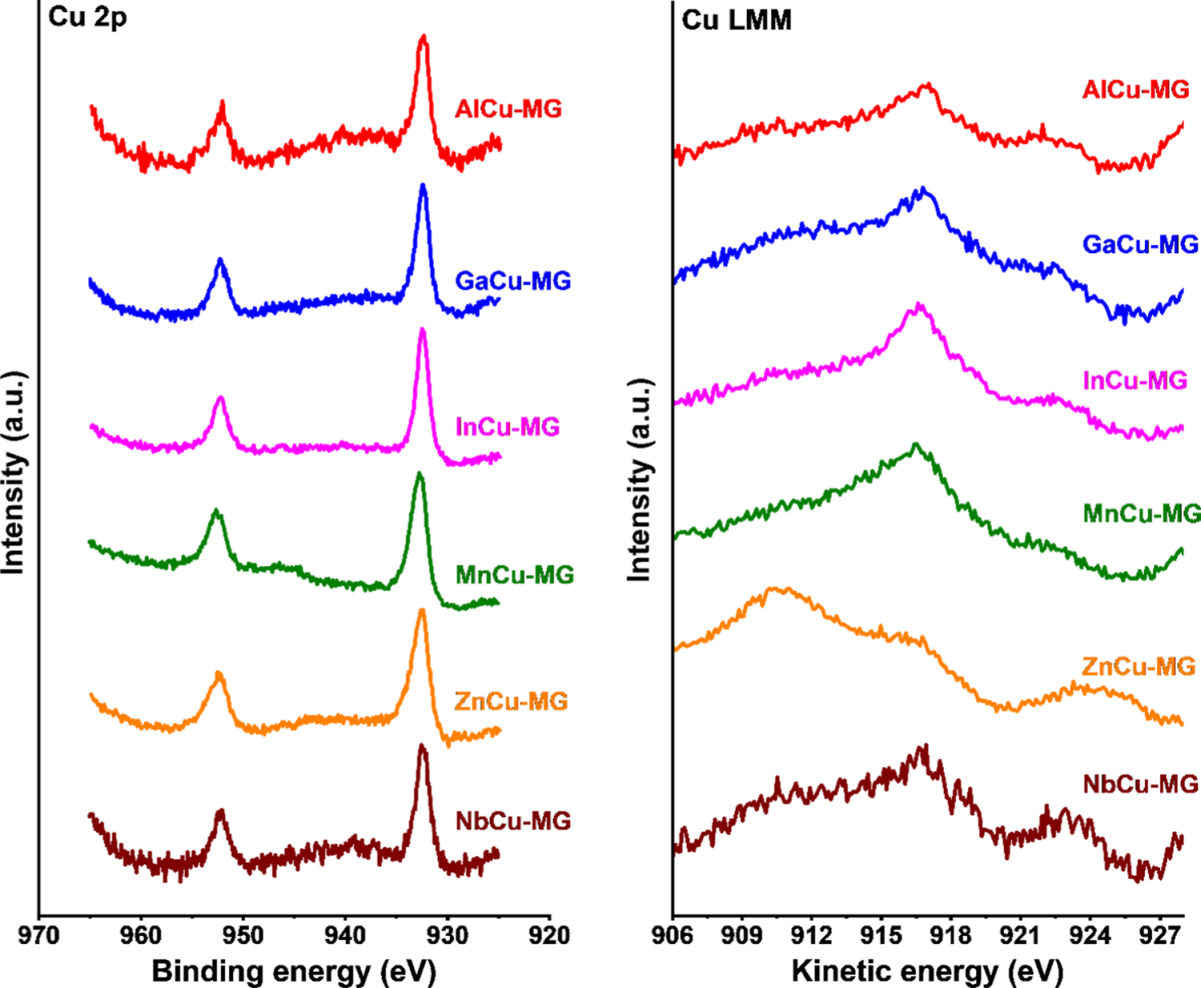
Cu 2p and Cu LMM regions of discharged MCu-MG catalysts after exposure to HTS conditions for 4 days at 25 bar.

The Cu 2p and Cu LMM regions of used MCu-doped catalysts are presented in Figure 9. The broad peaks at BE = 932 and 952 eV in the Cu 2p region can be attributed to Cu 2p_3/2_ and Cu 2p_1/2_ states, respectively.^47^ These values can be attributed to Cu^0^ or Cu^+^ species. The absence of Cu^2+^ is underpinned by the absence of a satellite feature at BE = ∼942 eV.^47^ Thus, it is not likely that Cu^2+^ is incorporated in the octahedral sites of ZnCu-MG. Inspection of the Cu LMM region (Figure 9) shows a peak at a kinetic energy of ∼917 eV^48^ in all MCu-MG catalysts, which implies that copper is mainly present as Cu^+^. The presence of Cu^+^ instead of Cu^0^ in activated HTS catalysts contradicts a recent *in situ* study performed by the Wachs group.^10^ In our earlier work on chromium–copper-doped catalysts, we also demonstrated that copper is present in the metallic state in activated catalysts.^6^ The observation of Cu^+^ in our used catalysts is likely the result of accidental oxidation during the shutdown procedure.

## Conclusions

Iron oxide phases in the precursor and activated WGS catalysts were promoted by a range of metals to establish their suitability to replace chromium. Emphasis was on establishing the structure and performance under close to practical conditions of the high-temperature WGS reaction. Similar to the nondoped hematite precursor, doping with chromium and gallium yielded hematite as the main precursor phase after calcination. Doping with aluminum, indium, manganese, zinc, and niobium resulted in a mixture of hematite and ferrihydrite, the latter in the form of relatively small crystallites. All calcined copper-promoted samples contained mainly ferrihydrite. The most suitable substituent for chromium in terms of catalytic performance is aluminum. Comparing the copper-copromoted catalysts under HTS conditions using an accelerated ageing protocol, it can be concluded that aluminum can replace chromium with nearly similar catalytic performance. The other substituents result in a significantly faster deactivation with the HTS performance decreasing in the order gallium > indium > manganese > zinc > niobium. The location of the dopants in the (predominantly) magnetite structure of the activated catalysts was further investigated by Mössbauer spectroscopy. Aluminum was incorporated in both tetrahedral and octahedral sites of magnetite in contrast to the chromium dopant, which exclusively substitutes octahedral iron. Aluminum doping did not affect the Fe^3+^/Fe^2+^ ratio, while chromium doping prevented Fe^2+^ formation during activation. The doping situation for the other catalysts varied with the doping metal. For indium and niobium, segregated promoter metal oxide phases were observed in addition to magnetite. Overall, the following generalizations can be made about the rational design of chromium-free HTS catalysts:

(1) The incorporation of trivalent ions of similar size to octahedral trivalent iron, such as aluminum, chromium, and gallium into the magnetite structure results in active and stable WGS catalysts, irrespective of the dopant incorporation in tetrahedral or octahedral positions. Aluminum doping leads to comparable activity and stability as chromium doping in a 4-day test under HTS conditions at 25 bar.
(2) Large trivalent ions, such as indium, do not remain in the iron oxide structure upon reduction of the Fe^3+^-oxide precursor to magnetite, resulting in segregated promoter oxide phases. Elements that can form separate iron-M-oxide phases under reducing conditions, such as niobium, are also unsuited to replace chromium in HTS catalysts.
(3) The incorporation of divalent ions with a tetrahedral site preference, such as zinc, results in complex structures where the charge imbalance is compensated by partial oxidation of Fe^2+^ in octahedral sites. No beneficial effect on WGS performance was observed for the Zn-promoted catalysts.
(4) The incorporation of divalent ions with an octahedral site preference, such as Mn^2+^, leads to a distortion of the octahedral Fe^3+^/Fe^2+^ redox couple because of the replacement of Fe^2+^ by Mn^2+^. Unlike substitution with trivalent ions with an octahedral site preference, Mn^2+^ substitution has a detrimental effect on HTS catalytic activity.

## Supporting Information Available

Magnified XRD patterns showing In_2_O_3_ and FeNb_2_O_6_ reflections; XPS spectra showing Al 2p, Ga 2p, In 3d, Mn 2p, Zn 2p, and Nb 3d regions of the used catalysts (PDF)
