# Supplementary material for: Substituting Chromium in Iron-Based Catalysts for the High-Temperature Water–Gas Shift Reaction
Source: ACS Catal. 2022 Oct 27;12(22):13838–52. doi: 10.1021/acscatal.2c03871 (PMC9679995; doi:10.1021/acscatal.2c03871)
Supplement: Supplementary file 1 — cs2c03871_si_001.pdf [file cs2c03871_si_001.pdf]

Supporting information

Substituting Chromium in Iron-Based Catalysts for the High-Temperature Water-Gas Shift Reaction

M.I. Ariëns[a,b], L.G.A. van de Water[c], A.I. Dugulan[a], E. Brück[a], E.J.M. Hensen[b*]

[a] Fundamental Aspects of Materials and Energy, Delft University of Technology, Mekelweg 15, 2629 JB Delft, The Netherlands

[b] Laboratory of Inorganic Materials and Catalysis, Department of Chemical Engineering and Chemistry, Eindhoven University of Technology, P.O. Box 513, 5600 MB Eindhoven, The Netherlands

[c] Johnson Matthey, PO Box 1, Belasis Avenue, Billingham, Cleveland, TS23 1LB, United Kingdom

*e-mail corresponding author: E.J.M.Hensen@TUE.nl

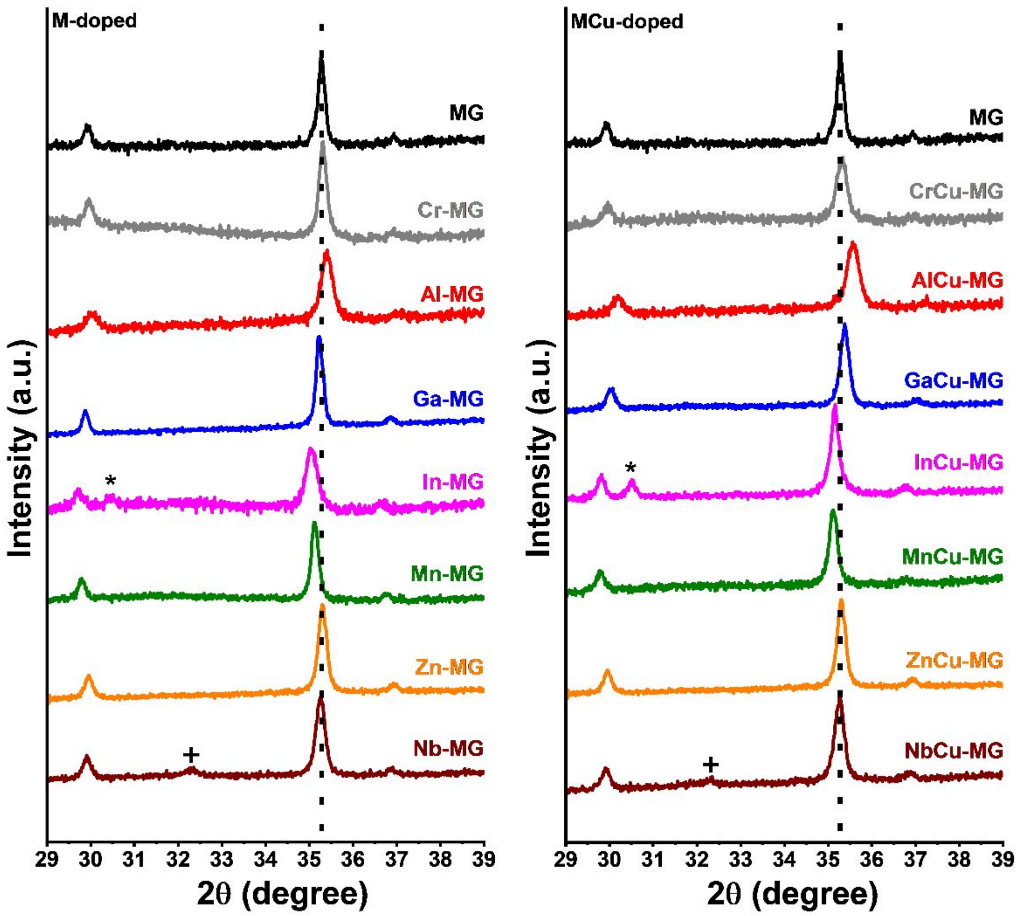

Figure S1. XRD patterns of discharged catalysts (M-MG, left; MCu-MG, right) recorded after exposure for 4 days at 25 bar. Magnetite (220) and (311) reflection at 2θ = ~30 and 2θ = ~35.3 respectively. In_2O_3 reflections are indicated with a (*), $FeNb_2O_6$ reflections with a (+). The MG reference in the MCu-MG column is non-doped magnetite.

The Al 2p region of the Al-MG and AlCu-MG catalysts are shown in Figure S2. The peak observed at BE = 74 eV points to the presence of Al^{3+} species [49] on the surface of the catalysts, in line with the formation of aluminium-doped magnetite in the Mössbauer spectra. From these spectra, we cannot deduce the presence of Al in different coordination environments. The Ga $2p_{3/2}$ peak at BE = ~1118 eV and the In $3d_{5/2}$ peak at BE = ~444 eV in the Ga 2p and In 3d spectra show the presence of these elements as the corresponding 3+-oxides [50,51]. We cannot distinguish their location in magnetite or as separate phases. It is difficult to judge the oxidation state from the Mn $2p_{3/2}$ peak at BE = 641 eV for Mn-MG and MnCu-MG (Fig. S2). However, the satellite at BE = 647 eV indicates that Mn is present in the 2+ oxidation state [52], which agrees with the Mössbauer data where Mn^{2+} replaced Fe^{2+} in octahedral positions in magnetite bulk. Zn is present as 2+ according to XPS [53], as expected. In the Nb-MG and NbCu-MG catalysts, the Nb $3d_{5/2}$ peak at BE = ~207 eV is in good agreement with literature report for

Nb^{5+} species [53]. No significant differences were observed between the M- and MCu-doped catalysts.

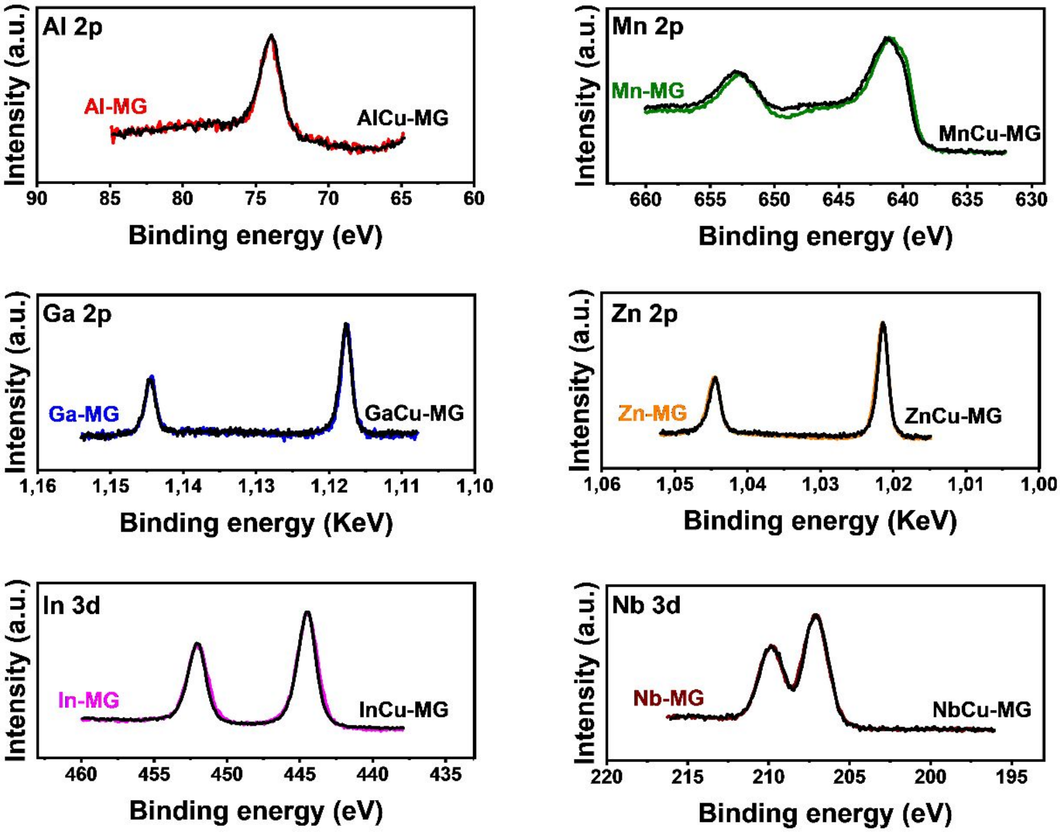

Figure S2. Al 2p, Ga 2p, In 3d, Mn 2p, Zn 2p, and Nb 3d regions of discharged M-MG and MCu-MG catalysts after exposure to HTS conditions for 4 days at 25 bar.
